# ß3 integrin modulates transforming growth factor beta induced (TGFBI) function and paclitaxel response in ovarian cancer cells

**DOI:** 10.1186/1476-4598-11-36

**Published:** 2012-05-28

**Authors:** David A Tumbarello, Jillian Temple, James D Brenton

**Affiliations:** 1 Cancer Research UK, Cambridge Research Institute, Robinson Way, Cambridge, CB2 0RE, United Kingdom; 2 Current Address: Cambridge Institute for Medical Research, Wellcome Trust/MRC Building, University of Cambridge, Hills Road, Cambridge, CB2 0XY, United Kingdom

**Keywords:** Chemotherapy, Cell adhesion, Ovarian cancer, Integrin receptor, Extracellular matrix

## Abstract

**Background:**

The extracellular matrix (ECM) has a key role in facilitating the progression of ovarian cancer and we have shown recently that the secreted ECM protein TGFBI modulates the response of ovarian cancer to paclitaxel-induced cell death.

**Results:**

We have determined TGFBI signaling from the extracellular environment is preferential for the cell surface αvß3 integrin heterodimer, in contrast to periostin, a TGFBI paralogue, which signals primarily via a ß1 integrin-mediated pathway. We demonstrate that suppression of ß1 integrin expression, in ß3 integrin-expressing ovarian cancer cells, increases adhesion to rTGFBI. In addition, Syndecan-1 and −4 expression is dispensable for adhesion to rTGFBI and loss of Syndecan-1 cooperates with the loss of ß1 integrin to further enhance adhesion to rTGFBI. The RGD motif present in the carboxy-terminus of TGFBI is necessary, but not sufficient, for SKOV3 cell adhesion and is dispensable for adhesion of ovarian cancer cells lacking ß3 integrin expression. In contrast to TGFBI, the carboxy-terminus of periostin, lacking a RGD motif, is unable to support adhesion of ovarian cancer cells. Suppression of ß3 integrin in SKOV3 cells increases resistance to paclitaxel-induced cell death while suppression of ß1 integrin has no effect. Furthermore, suppression of TGFBI expression stimulates a paclitaxel resistant phenotype while suppression of fibronectin expression, which primarily signals through a ß1 integrin-mediated pathway, increases paclitaxel sensitivity.

**Conclusions:**

Therefore, different ECM components use distinct signaling mechanisms in ovarian cancer cells and in particular, TGFBI preferentially interacts through a ß3 integrin receptor mediated mechanism to regulate the response of cells to paclitaxel-induced cell death.

## Background

Ovarian cancer is the deadliest gynaecological cancer in women with the development of chemotherapeutic drug resistance being the major obstacle to successful treatment. Recent data suggests that the extracellular matrix (ECM) can directly modulate cell sensitivity to both platinum- and taxane-based drug treatment therapies [1-4]. Also, as the ECM regulates other key aspects of cell behaviour including growth control, cell migration, survival, and gene expression [5], it represents an important target in designing treatment therapies.

We have shown that the secreted extracellular matrix protein, TGFBI (transforming growth factor beta induced), is a critical component of the ovarian cancer tumor microenvironment that sensitizes cells to paclitaxel-induced cell death by stabilizing microtubules via integrin-mediated activation of focal adhesion kinase (FAK) and the Rho family GTPase RhoA [1]. TGFBI has been suggested to have both tumor suppressor and tumor promoting properties, depending on the cancer of origin [6]. Specifically, TGFBI has been shown to be underexpressed in breast [7], ovarian, and lung cancer [8]; and overexpressed in clear cell renal carcinoma [9], pancreatic cancer [10], and colorectal cancer [11]. In addition, mice lacking *Tgfbi* show spontaneous tumor formation, further supporting a potential tumor suppressor function [12]. Interestingly, loss of TGFBI expression is associated with centrosome duplication and chromosomal instability, both causal factors associated with carcinogenesis and drug resistant phenotypes [1,12,13]. However, the mechanism by which extracellular TGFBI mediates these effects is unclear.

Structurally, TGFBI contains an amino-terminal signal peptide sequence necessary for secretion into the extracellular environment, a cysteine-rich EMI domain similar to regions found in proteins of the EMILIN family, along with four highly conserved fasciclin I (FAS I) domains and a carboxy-terminal Arginine-Glycine-Aspartic Acid (RGD) motif. Various heterodimeric integrin receptor combinations mediate interactions with TGFBI and its RGD and FAS I domains [14-16]. Specifically, corneal epithelial cell adhesion to TGFBI is predominantly mediated by the α3ß1 integrin heterodimer [14], while in endothelial cells the αvß3 integrin heterodimer is dominant [15]. Furthermore, TGFBI can bind many ECM proteins including Collagen type I, II, IV, and VI [17-19], fibronectin [20], periostin [21], laminin [18], as well as the proteoglycans biglycan and decorin [22]. The FAS domains are highly conserved and three human proteins, TGFBI, periostin, and stabilin, contain these motifs [23].

Periostin is a paralogue of TGFBI and is also a TGFß1-inducible secreted protein. Both TGFBI and periostin have been implicated in ovarian cancer [1,24]. Periostin is secreted by ovarian cancer, similar to TGFBI, and promotes integrin-mediated cell motility [24]. However, although they have similar domain structure, very little is known as to whether their function is complementary or antagonistic. Periostin shares with TGFBI an EMI domain and four highly conserved FAS I domains. However, it differs in having an extended carboxy-terminus, which does not contain the RGD motif [25,26]. Interestingly, recent data suggests periostin and TGFBI interact through their amino-terminal EMI domains and may have a proactive role in the pathogenesis of corneal dystrophy [21]. Additionally, periostin contributes to metastasis in both pancreatic and colon cancer due to augmentation of PI3K/Akt signaling [27,28] and it has been suggested to be a critical component of metastatic colonization [29]. Therefore, evaluating the mechanism of TGFBI and periostin function in ovarian cancer cells may shed light on their relationship and function during ovarian carcinogenesis.

Although TGFBI has been shown to signal through multiple integrin heterodimeric receptors, the predominant signaling pathways and the relationship to other ECM components in ovarian cancer is unknown. It has been shown that fibronectin-integrin signaling could protect breast cancer cells against paclitaxel-induced cell death [30]. Since this contrasts to the function of TGFBI in ovarian cancer [1], there lacks a clear understanding of the differential signaling that occurs upon engagement of the cell surface with various ECM components. Importantly, previous reports have suggested that cross-talk between different integrin receptors can modulate the response to their respective ECM ligand [31-33].

To understand the function of TGFBI in ovarian cancer and the role of TGFBI-integrin interactions in mediating paclitaxel sensitivity, we therefore delineated the primary domains of TGFBI that are important in mediating the interaction with ovarian cancer cells and the key receptors necessary for this process.

## Methods

### Antibodies and reagents

Paclitaxel was purchased from Sigma-Aldrich, cat. no. T7402 (Dorset, UK). The GRGDSP peptide was purchased from Merck Chemicals Ltd. (Nottinghamshire, UK) and the ERGDEL peptide was custom produced by Sigma Genosys (Haverhill, UK). Human plasma fibronectin was purchased from Millipore (Watford, UK) and human vitronectin was purchased from R&D systems Europe Ltd. (Abingdon, UK). Affinity purified polyclonal antibody directed against TGFBI was produced by immunizing rabbits with a C-terminal peptide of human TGFBI (aa 498–683). All antibody production was performed in collaboration with Cambridge Research Biochemicals (Cleveland, UK). TGFBI polyclonal antiserum was a kind gift from Dr. Ching Yuan (University of Minnesota, Minnesota, USA). Alpha-tubulin antibody was purchased from Sigma-Aldrich. The periostin polyclonal antibody was purchased from BioVendor Laboratory Medicine Inc. (Czech Republic) and the periostin monoclonal antibody (clone 345613) from R&D Systems Europe Ltd. Akt phospho-S473 and pan-Akt polyclonal antibodies were purchased from Cell Signaling. Fibronectin, ILK, and FAK phospho-Y397 monoclonal antibodies were purchased from BD Biosciences (Oxford Science Park, Oxford, UK). Alexa Fluor 568-phalloidin was purchased from Invitrogen (Inchinnan Business Park, Paisley, UK). ß3 integrin polyclonal antibody was purchased from Santa Cruz (Santa Cruz, California) and ß1 integrin polyclonal antibody was purchased from Cambridge Bioscience (Cambridge, UK). Integrin blocking antibodies against ß1 integrin (clone P5D2), αvß3 (clone LM609), and αvß5 (clone P1F6) were purchased from Millipore. Syndecan-1 monoclonal antibody was purchased from Serotec (Oxford, UK) and Syndecan-4 polyclonal antibody was purchased from R&D Systems Europe Ltd.

### Cell culture

The ovarian cancer SKOV3 cell line was maintained in RPMI media supplemented with 10% (v/v) heat-inactivated FBS, 50 units/ml penicillin, and 50 μg/ml streptomycin. The ovarian cancer PEO1 cell line was maintained in DMEM/F12 (50:50) supplemented with 10% (v/v) heat inactivated FBS, 50 units/ml penicillin, and 50 μg/ml streptomycin. NIH 3 T3 cells were maintained in DMEM supplemented with 10% (v/v) heat inactivated FBS, 50 units/ml penicillin, and 50 μg/ml streptomycin. All cell lines were verified by short tandem repeat genotyping. Lentivirus expressing individual shRNA targeted against ß1 integrin, ß3 integrin, TGFBI, and fibronectin were purchased from Sigma-Aldrich Mission® shRNA library. Cells were infected at an MOI of 10 and subsequently stable pools of cells were selected in Puromycin. Syndecan-1 siGenome SMARTpool® siRNA, syndecan-4 siGenome SMARTpool®, ß1 integrin ON-TARGETplus® pool, ß3 integrin ON-TARGETplus® pool, and siGenome® non-target control #2 siRNA were purchased from Perbio (Northumberland, UK). siRNA transfections were performed using Lipofectamine 2000 (Invitrogen, Inchinnan Business Park, Paisley, UK) according to manufacturer’s instructions.

### Western blot

Cell lysates were harvested in RIPA buffer (1% Triton X-100, 0.1% SDS, 1% DOC, 10 mM Tris–HCl pH 7.4, 150 mM NaCl, 5 mM EDTA, 10 μg/ml leupeptin, and 1 mM Na_3_VO_4_). Lysates were cleared by centrifugation at 14,000xg at 4°C. Protein content was quantified by the BioRad D_c_ Protein Assay (Hertfordshire, UK). Following the addition of 2X SDS-sample buffer and boiling, samples were loaded onto 7.5-10% SDS-PAGE gels and transferred to PVDF (Fisher Scientific UK, Leicestershire, UK). Membranes were blocked with either 5% non-fat dry milk or 3% BSA, probed with the indicated antibodies, and visualized following the addition of HRP conjugated secondary antibodies (Dako UK Ltd., Cambridgeshire, UK) and incubation with enhanced chemiluminescence (GE Healthcare UK Ltd., Buckinghamshire, UK). Western blots were either directly reprobed or parallel Western blots were performed on the same cell lysates for alpha-tubulin loading controls.

### Cell surface biotinylation

Cells were washed in cold PBS, incubated 30 minutes with 0.2 mg/ml EZ-Link Sulfo-NHS-Biotin (Fisher Scientific UK Limited, Loughborough, UK) on ice. After 30 minutes, cells were washed two times in cold PBS and lysed in immunoprecipitation buffer (200 mM NaCl, 75 mM Tris pH7.4, 7.5 mM EDTA, 7.5 mM EGTA, 1.5% Triton-X 100 and 0.7% NP-40 with protease inhibitor cocktail). Lysate was cleared at 14,000 xg for 10 minutes at 4°C and the resulting supernatant was incubated with anti-β3 integrin/CD61 antibody (clone VI-PL2; BD Biosciences) overnight at 4°C, followed by addition of 15 μL of 50 mg/ml Protein A-sepharose and incubation at 4°C for 1 hour. Beads were washed four times in lysis buffer, followed by addition of 2X SSB, and samples were run under non-reducing conditions on 7.5% SDS-PAGE. Western blot analysis was performed with HRP-conjugated streptavidin (Fisher Scientific UK Limited).

### Recombinant protein production

The pET27 TGFBI plasmid utilized for recombinant protein production in bacteria was a kind gift from Dr. Ching Yuan (University of Minnesota, Minnesota, USA). Both recombinant TGFBI and periostin were engineered with a carboxy-terminal His-tag. Periostin cDNA was a kind gift from Dr. Nick Lemoine (Barts, London, UK). Periostin cDNA, lacking the amino-terminal signal peptide, was cloned into the pET27 vector for subsequent production of bacterial expressed recombinant protein. Deletion constructs were made by PCR addition of NheI and NdeI unique restriction sites for subsequent cloning into the pET27 vector. Site-directed mutagenesis was performed on pET27 TGFBI to produce an amino acid RGD to RAE substitution using the oligonucleotide primer 5’-agacctcaggaaagagcggaggaacttgcagactctg-3’ and an amino acid YH to SR substitution using the oligonucleotide primer 5’-gaacttgccaacatcctgaaagccgccattggtgatgaaatcctgg-3’. All constructs were verified by sequencing. All recombinant proteins were produced in Rosetta BL21 (DE3) E.coli (Merck, Nottingham, UK) and either purified from an insoluble fraction [34] for full-length TGFBI and periostin or from a soluble fraction [35] utilizing Ni-NTA agarose beads (Merck). Refolding of purified full-length TGFBI and periostin was performed by buffer exchange through a PD10 Desalting Column (GE Healthcare, Buckinghamshire, UK) into 10 mM Tris–HCl pH 7.4, 0.5 M Arginine-HCl, and 10% Glycerol solution.

### Adhesion assay

96-well or 24-well tissue culture treated plastic dishes were incubated overnight at 37°C with 20 μg/ml of recombinant protein diluted in PBS. Dishes were subsequently washed with PBS, blocked with 3% BSA for 1 hour at 37°C, followed by washing with PBS and SF media containing 0.1% BSA. Cells were collected, washed once with growth media, washed twice with serum-free media containing 0.1% BSA, and incubated in serum-free media containing 0.1% BSA for 1 hour at 37°C in suspension. Cells were plated on uncoated, poly-L-lysine, or matrix-coated dishes for indicated time periods. Adherent cells were subsequently washed once with PBS, fixed in methanol, and stained with Giemsa (Fisher Scientific UK). Stain was eluted with 10% acetic acid and an absorbance reading was obtained at 540 nm. To account for non-specific adhesion, values from uncoated wells were subtracted from all experimental values. All experiments were performed in triplicate. Due to technical variability of raw values between replicate experiments, data were represented as percent adhesion to control. All statistics were performed in GraphPad Prism® using either one- or two-way Anova along with Bonferroni’s multiple comparison test when appropriate. Error bars represent standard deviation. Bright-field images were taken with a DS-Fi1 CCD camera and processed with Adobe Photoshop® CS2.

### Apoptosis and viability assays

For apoptosis analysis, cells plated on uncoated tissue culture dishes were treated with varied concentrations of Paclitaxel or DMSO vehicle control diluted in complete growth media. Following incubation at 37°C for 24 hours, both adherent and floating cells were harvested and washed in cold PBS. The TACS Annexin-V Apoptosis kit (R&D systems Europe Ltd.) was performed according to manufacturer’s instructions. 10,000 cell events were recorded on a BD FACS Calibur and data was analyzed with FlowJo 8.8.4 flow cytometry analysis software (Tree Star Inc., Ashland, Oregon, USA). Results are represented as the percentage of early apoptotic events (Annexin-V positive, propidium iodide negative) compared to total events and error bars represent standard deviation. For cell viability analysis, cells were transiently transfected with siRNA prior to replating on white 96-well tissue culture dishes. Cells were treated with vehicle (DMSO) or increasing concentrations of Paclitaxel for 48 hours prior to administration of the Cell Titre Glo® Luminescent cell viability reagent as per manufacturer’s instructions (Promega UK, Southampton, UK). Results were normalized to a DMSO treated control and the experiment was performed in triplicate. Error bars represent standard deviation and a one-way anova along with a Bonferroni multiple comparison test was performed.

### Immunofluorescence microscopy

Cells were fixed in 3.7% Formaldehyde in PBS for 8 minutes and permeabilized with 0.2% Triton X-100 for 2 minutes. Fixed cells were incubated with primary antibody in TBS containing 1% BSA at 37°C for 1.5 hours, washed in TBS, incubated with either Alexa Fluor® 488 or 568 secondary antibodies (Invitrogen) in TBS containing 1% BSA, washed in TBS, and mounted in Fluorsave (Calbiochem). For live cell immunostaining with anti-αvß3 integrin antibody (clone LM609), cells were first washed into CO_2_-independent medium supplemented with 2% FBS, next incubated in primary antibody for 20 minutes, followed by incubation with Alexa Fluor 488 antibody for 20 minutes. Cells were washed in PBS and fixed for 2 minutes in ice-cold methanol. Nuclei were stained with Hoechst and coverslips were mounted in Fluorsave. Images were captured on a Leica Tandem SP5 confocal microscope (Leica-Microsystems, Milton Keynes, UK) or a Zeiss Axioplan epifluorescence microscope equipped with a Hamamatsu ORCA-R2 CCD camera driven by Simple PCI software (Carl Zeiss MicroImaging Inc.) and processed with Adobe Photoshop® CS2. Image analysis of cell surface integrin immunostaining was performed using ImageJ software. Briefly, the integrated intensity of integrin immunostaining was calculated and due to technical variability between replicate experiments, values were normalized to control and represented as the percent change in fluorescence intensity. The data represents at least 100 individual cells taken from two independent experiments.

Bright-field time lapse video microscopy was performed using a Nikon TE2000 PFS microscope equipped with a DS-Fi1 CCD camera. Cells were plated on a matrix-coated ibidi 35 mm μ-dish, low (Thistle Scientific, Glasgow, UK) and images were acquired using a 10X objective every 2 minutes for 6 hours using NIS elements software (Nikon Instruments Europe) in a temperature controlled and 5% CO_2_ maintained environment.

## Results

### Recombinant TGFBI and periostin support adhesion of ovarian cancer cells and stimulate Akt phosphorylation

**Figure 1:**
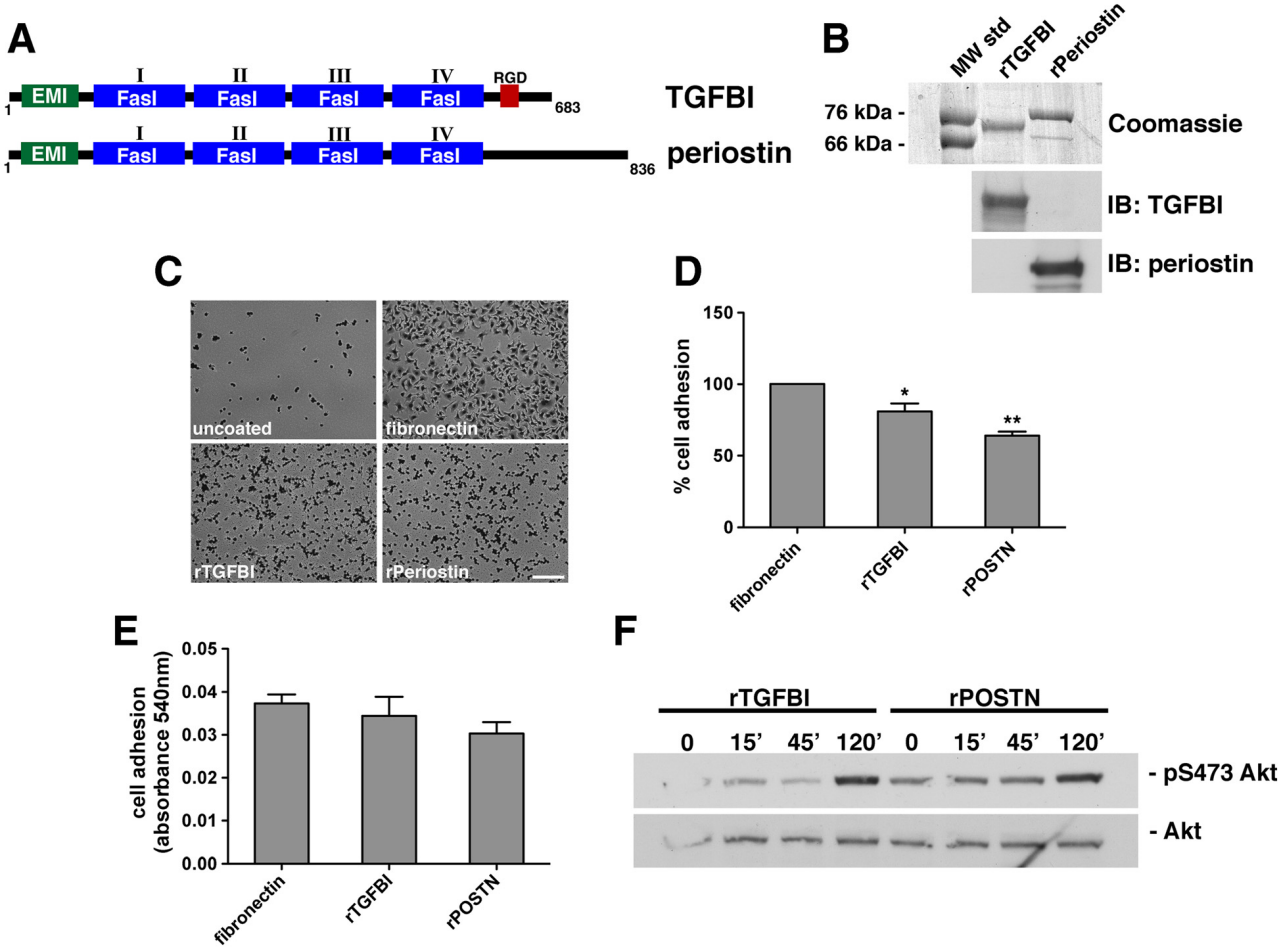
**Recombinant TGFBI and periostin support adhesion of ovarian cancer cells and stimulate Akt phosphorylation.A,** Schematic representation of the domain structure of TGFBI and periostin. Both TGFBI and periostin contain conserved Fasciclin I and EMI domains, while only TGFBI contains an RGD motif. **B,** Purified bacterially expressed recombinant TGFBI (rTGFBI) and recombinant Periostin (rPOSTN). Coomassie brilliant blue stained SDS-PAGE of purified rTGFBI and rPOSTN and Western blot (IB) probed with specific antibodies against TGFBI and periostin. **C,** Bright-field images of SKOV3 adhesion to uncoated, fibronectin, rTGFBI, or rPOSTN coated tissue culture plastic. **D,** Results of three independent adhesion experiments were normalized to poly-L-lysine and are represented as percent of fibronectin control, *p < 0.05, **p < 0.01. **E,** NIH3T3 cells were replated on tissue culture wells coated with 10 μg/ml of fibronectin, rTGFBI, or rPeriostin and allowed to adhere for 30 minutes. Results of two independent experiments were normalized to poly-L-lysine and represented as percent of fibronectin control. **F,** Western blot analysis of NIH3T3 cell lysates following stimulation with 10 μg/ml of rTGFBI or rPeriostin in serum-free media for indicated time points. The membrane was probed with antibodies specific to the phosphorylated Serine 473 amino acid residue of Akt and pan-Akt antibodies were utilized as loading control.

Both TGFBI and periostin contain conserved motifs shown to mediate binding to the integrin receptor family. However, although TGFBI and periostin retain the four conserved fasciclin I domains, periostin contains a longer carboxy-terminus lacking an RGD motif, which is present in TGFBI (Figure 1A). Importantly, the RGD motif has been implicated in integrin receptor binding and has been shown to be necessary for cell adhesion to various extracellular proteins, including fibronectin [36].

We first compared the functions of TGFBI and periostin on ovarian cancer cells. Firstly, recombinant TGFBI (rTGFBI) and periostin (rPOSTN) were produced from bacteria and expression was verified by SDS-PAGE and Western blot (Figure 1B). To validate the functions of the recombinant proteins and to determine whether ovarian cancer cells have differential binding to both matrices, the SKOV3 ovarian cancer cell line was used in adhesion assays. SKOV3 cells were capable of adhering and spreading on both recombinant TGFBI and periostin, although adhesion to periostin was less than TGFBI or fibronectin (Figure 1C, 1D; Additional file 1: Movie S1 and Additional file 2: Movie S2).

Previous reports have suggested periostin and TGFBI are capable of stimulating Akt phosphorylation [27,28,37]. We evaluated the potential biochemical differences in Akt phosphorylation following interaction of cells with either rTGFBI or rPOSTN. As SKOV3 and other ovarian cancer cell lines have constitutive activation of Akt we used NIH 3T3 cells, which are capable of supporting adhesion to both rTGFBI and rPOSTN (Figure 1E), and have low basal levels of Akt phosphorylation. Both rTGFBI and rPOSTN were capable of phosphorylating Akt at serine 473 in NIH 3T3 cells (Figure 1F).

### Integrin subunit expression influences the extent of TGFBI adhesion

**Figure 2:**
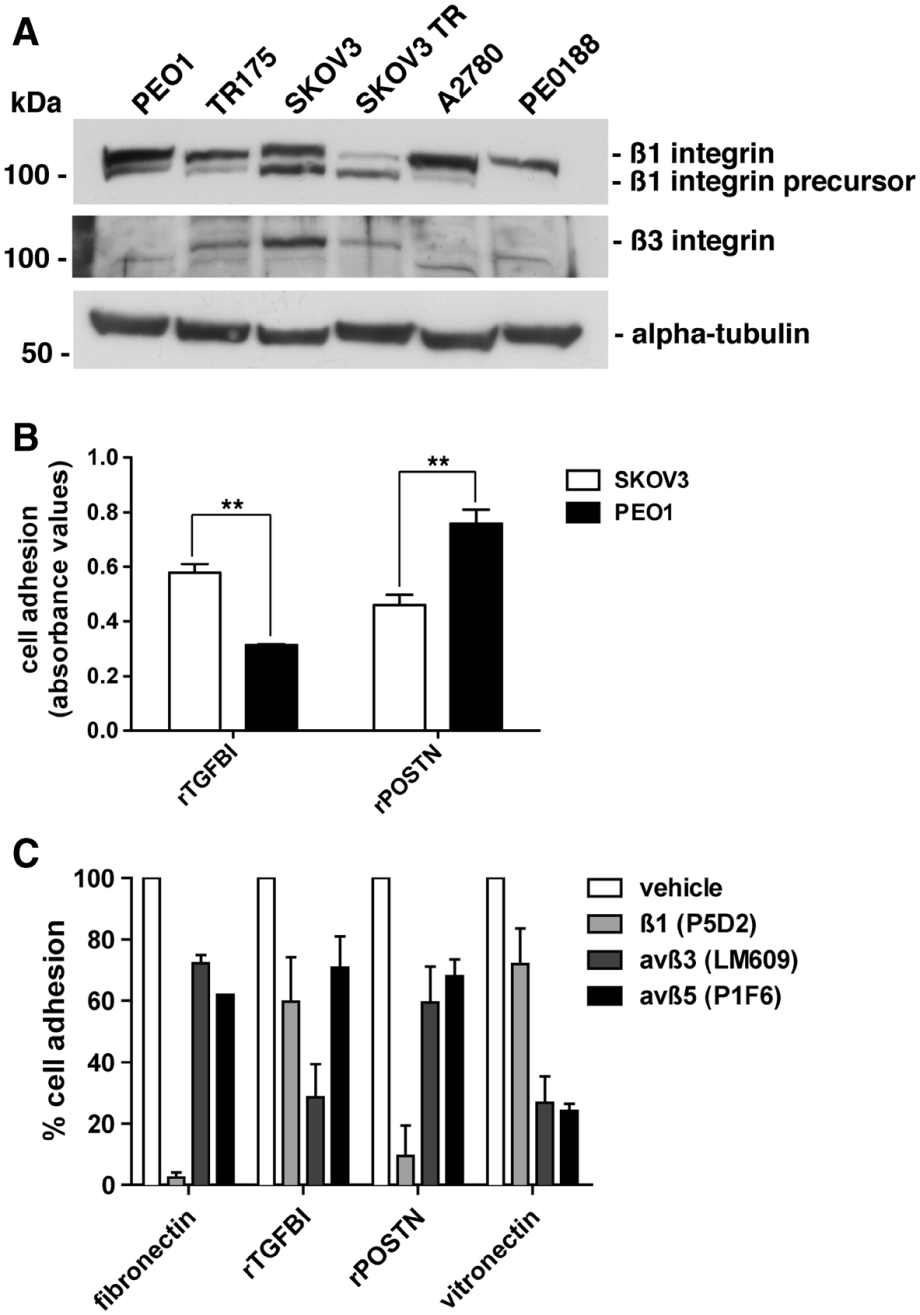
**Integrin subunit expression influences the extent of TGFBI adhesion.A,** Western blot analysis of RIPA soluble lysates from a panel of ovarian cancer cell lines probed with antibodies against the indicated proteins. SKOV3 TR cells are an *in vitro*-derived taxol resistant derivative of the SKOV3 parental line. **B,** Relative adhesion of SKOV3 and PEO1 ovarian cancer cell lines to rTGFBI and rPeriostin. Results of two independent experiments are represented as absorbance at 540 nm, **p < 0.01. **C,** SKOV3 cell adhesion to fibronectin, rTGFBI, rPOSTN, and vitronectin coated tissue culture plastic in the presence of vehicle or the indicated integrin blocking antibodies. Results of at least three independent experiments were normalized to poly-L-lysine and represented as percent of vehicle treated control.

Primary ovarian tumor samples and ovarian cancer cell lines have been shown to have variable expression of different integrin subunits [38]. This variable integrin expression profile may influence cell interactions with the ECM. We characterized a panel of six ovarian cancer cell lines for ß1 and ß3 integrin subunit expression. Western blot analysis indicated ubiquitous expression of ß1 integrin while ß3 integrin expression was limited to the TR175, SKOV3 and the *in vitro* derived taxol-resistant SKOV3 TR cell lines (Figure 2A; Additional file 3: Figure S1). SKOV3 cells (ß1 and ß3 integrin positive) preferentially bound to recombinant TGFBI, while PEO1 cells (ß1 integrin positive, ß3 integrin negative) preferentially bound to recombinant periostin (Figure 2B). To further evaluate the specificity of TGFBI and periostin for β1 and β3 integrin heterodimers we used function blocking integrin antibodies and adhesion assays with SKOV3 cells. TGFBI predominantly signalled through an αvß3 integrin-mediated mechanism, periostin and fibronectin preferentially signalled through a ß1 integrin-mediated mechanism, and vitronectin primarily utilized αvß3 and αvß5 integrins (Figure 2C). To ensure that the effect on TGFBI was ß3 integrin specific, we used the ß3 integrin null cell line, PEO1, which resulted in no difference in adhesion to rTGFBI following preincubation with an αvß3 integrin function blocking antibody ( Additional file 4: Figure S2).

### Loss of ß1 integrin expression stimulates cell adhesion and spreading to rTGFBI in ovarian cancer cells

**Figure 3:**
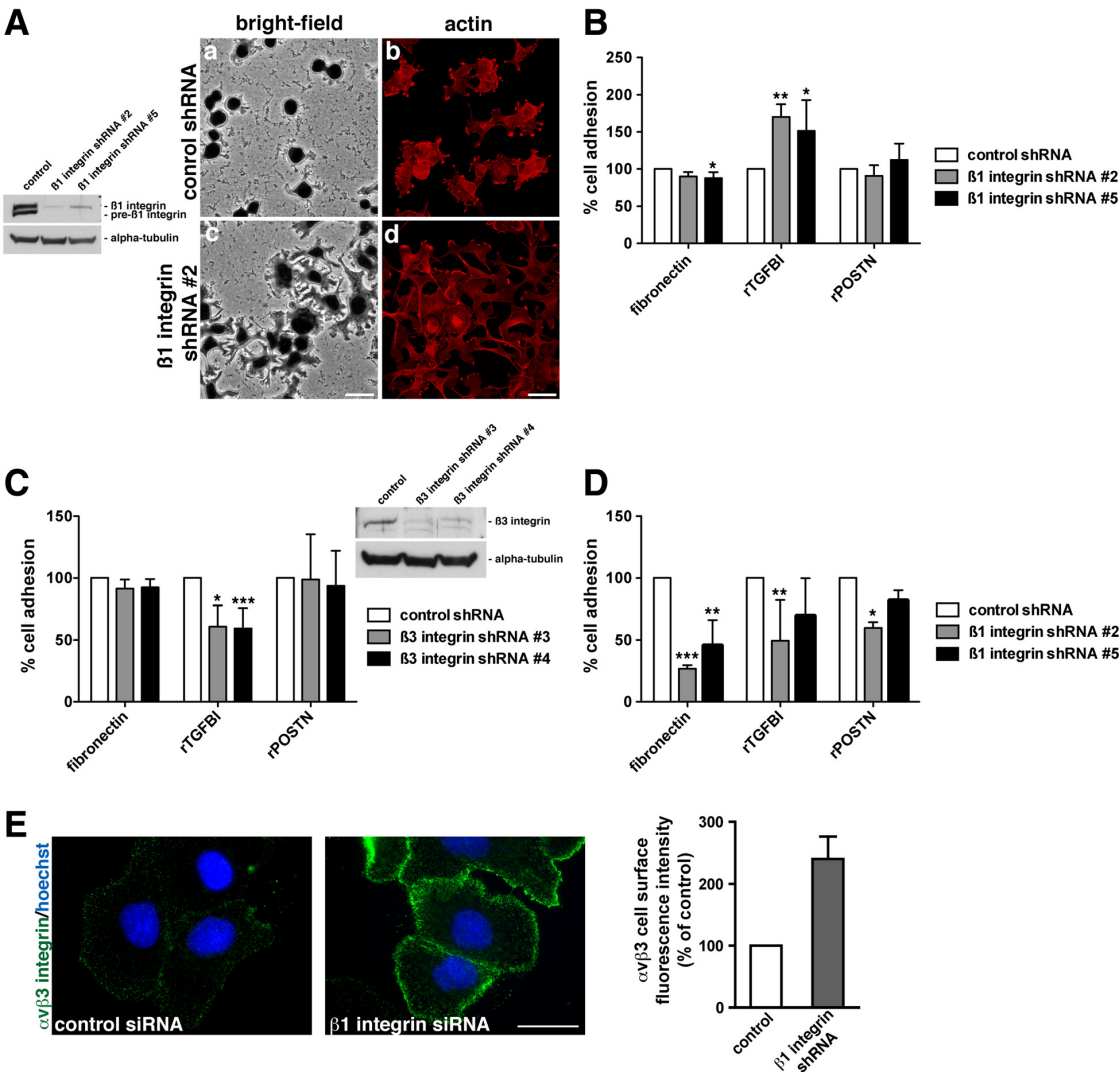
**Loss of ß1 integrin expression stimulates cell adhesion and spreading to rTGFBI in ovarian cancer cells.A,** SKOV3 cells infected with Lentivirus expressing shRNA against ß1 integrin. Western blot analysis of RIPA soluble lysates utilizing antibodies against ß1 integrin or alpha-tubulin. Bright-field (a,c) and confocal immunofluorescence microscopy (b,d) images of control non-target shRNA (a,b) or ß1 integrin shRNA (c,d) treated cells following adhesion to rTGFBI. Rhodamine-phalloidin was utilized to visualize the actin cytoskeleton. Scale bar = 40 μm (confocal), scale bar = 50 μm (bright-field). **B,** SKOV3 cells were either control non-target shRNA or ß1 integrin shRNA treated followed by incubation on either fibronectin, rTGFBI, or rPeriostin coated tissue culture plastic for 30 minutes. Results of three independent experiments were normalized to poly-L-lysine and represented as percent of non-target control shRNA on each matrix protein. Significance of *p < 0.05 and **p < 0.01 is compared to control shRNA. **C,** SKOV3 cells infected with Lentivirus expressing shRNA against ß3 integrin. Cells expressing either non-target shRNA or ß3 integrin shRNA were replated on fibronectin, rTGFBI, or rPOSTN coated wells and allowed to adhere for 30 minutes. Results of three independent experiments were normalized to poly-L-lysine and represented as percent of non-target control shRNA on each matrix. Significance of *p < 0.05 and ***p < 0.001 is compared to control shRNA. **D,** PEO1 cells expressing either non-target shRNA or ß1 integrin shRNA were replated on fibronectin, rTGFBI, or rPOSTN coated wells and allowed to adhere for 1 hour. Results of three independent experiments were normalized to poly-L-lysine and represented as percent of non-target control shRNA on each matrix. Significance of *p < 0.05, **p < 0.01, and ***p < 0.001 is compared to control shRNA. **E,** SKOV3 cells following siRNA transfection against ß1 integrin or non-target control were processed for live cell immunostaining against the αvß3 integrin heterodimer using the LM609 antibody (green). Hoechst stain was utilized to visualize the nuclei (blue). Scale bar 40 μm. Quantitation of live cell immunostained avß3 integrin heterodimers was achieved using ImageJ software. All experiments were performed in duplicate and analysis was performed on greater than 100 cells/experiment. Results are represented as αvß3 integrin cell surface fluorescence intensity compared to control siRNA cells.

The interaction of TGFBI with cell surface integrin receptors is complex, and is likely cell-type specific [6]. Variable expression of different integrin subunits in ovarian cancer has been reported, including upregulation of ß3 integrin expression and its association with metastasis [39,40]. Thus, we evaluated the effects of dynamic modulation of the ß1 and ß3 integrin subunits during adhesion to fibronectin, TGFBI, and periostin. To assess the specificity of the TGFBI interaction with specific cell surface integrin heterodimers, short hairpin RNAs (shRNA) targeting either ß1 or ß3 integrin were utilized to delineate their individual contributions. SKOV3 cells were infected with different Lentiviruses expressing two separate shRNA targets to ß1 integrin or ß3 integrin as well as a non-target control shRNA, and stable pools of cells were selected with puromycin. All shRNA targets to ß1 and ß3 integrin suppressed protein expression as assessed by Western blot (Figure 3A3C). Knockdown of ß1 integrin expression, using two distinct shRNA target sequences in SKOV3 cells, stimulated their adhesion and spreading on recombinant TGFBI, while having a minimal effect on recombinant periostin (Figure 3A3B). In contrast, loss of ß3 integrin expression specifically suppressed adhesion to recombinant TGFBI (Figure 3C). Furthermore, in the PEO1 cell line, which lacks ß3 integrin expression (Figure 2A; Additional file 3: Figure S1), reduced adhesion to rTGFBI was observed following suppression of ß1 integrin expression, suggesting ß3 integrin expression is necessary for the increased adhesion associated with SKOV3 cells (Figure 3D). This was confirmed by a reduction in adhesion of the ß1 integrin shRNA expressing SKOV3 cells to rTGFBI after incubation with an αvß3 integrin-blocking antibody ( Additional file 5: Figure S3).

**Figure 4:**
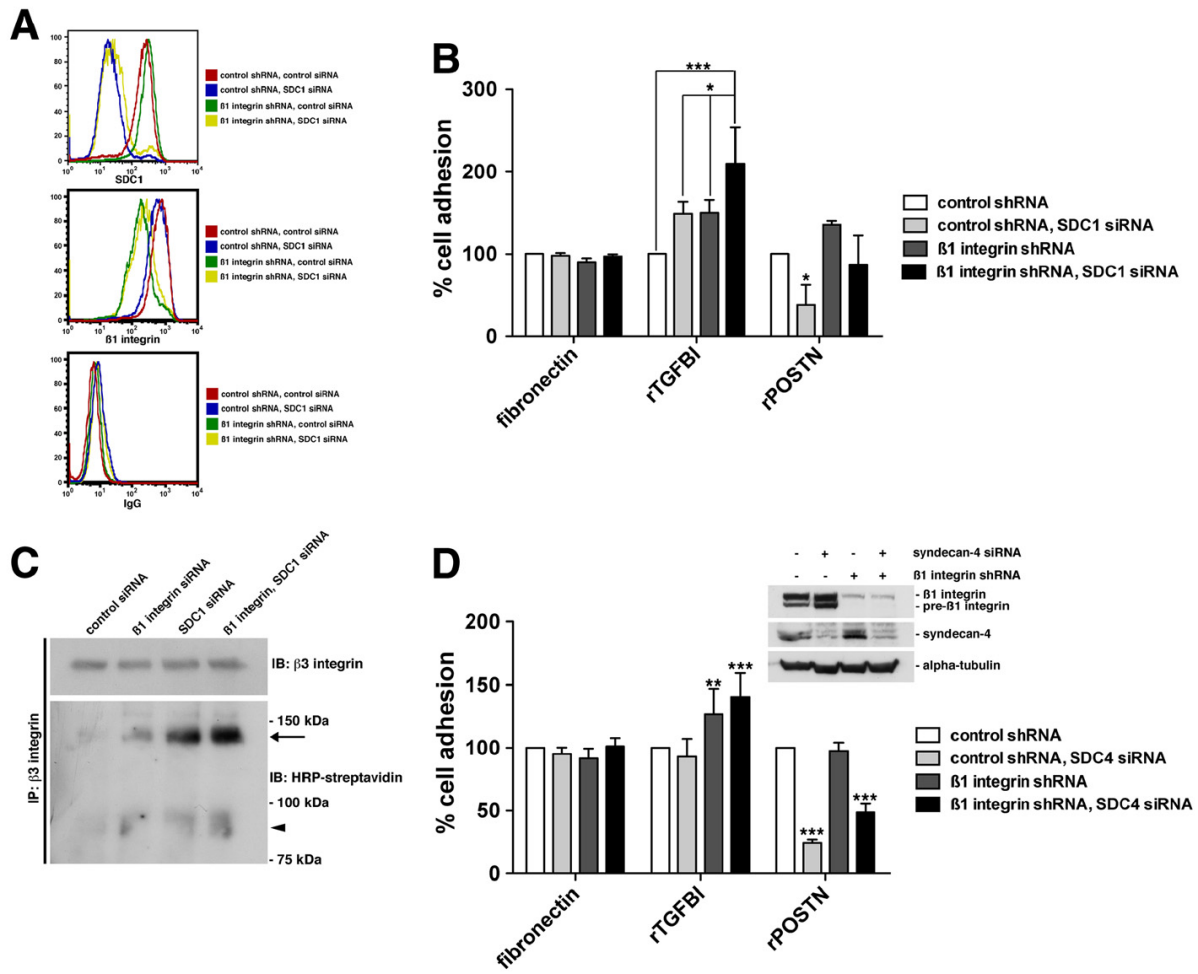
**Suppression of Syndecan-1 expression synergizes with the suppression of ß1 integrin expression to stimulate SKOV3 adhesion to rTGFBI.A,** SKOV3 cells with stable expression of control non-target or ß1 integrin shRNA were transfected with control non-target siRNA or Syndecan-1 siRNA. Flow cytometric analysis of ß1 integrin and Syndecan-1 cell surface protein expression was performed. **B,** Cells were replated on either fibronectin, rTGFBI, or rPOSTN coated tissue culture wells and allowed to adhere for 30 minutes. Results were normalized to poly-L-lysine and represented as percent of non-target control shRNA, *p < 0.05, ***p < 0.001. **C,** Cell surface biotinylation and immunoprecipitation of ß3 integrin from SKOV3 cells expressing either non-target control, ß1 integrin, SDC-1, or ß1 integrin/SDC-1 siRNA. Western blot analysis was performed against ß3 integrin or against biotin using HRP-streptavidin. Arrow indicates αv integrin subunit and arrowhead indicates ß3 integrin subunit. **D,** SKOV3 cells with stable expression of control non-target or ß1 integrin shRNA were transfected with control non-target siRNA or Syndecan-4 siRNA. Western blot analysis was performed on RIPA soluble lysates and the membrane was probed with antibodies specific to the indicated proteins. Cells were replated on either fibronectin, rTGFBI, or rPOSTN coated tissue culture wells and allowed to adhere for 30 minutes. Results were normalized to poly-L-lysine and represented as percent of non-target control shRNA. Significance of **p < 0.01 and ***p < 0.001 is compared to control shRNA.

Since suppression of ß1 integrin expression had no effect on ß3 integrin expression (data not shown), we next wanted to determine whether there was a modulation in cell surface expression of ß3 integrin. Following transfection of SKOV3 cells with ß1 integrin siRNA, live cell immunostaining revealed increased cell surface expression of the αvß3 integrin heterodimer in ß1 integrin siRNA treated compared to control non-target siRNA treated cells (Figure 3E). This cortically arranged immunostaining pattern was verified when evaluating focal adhesions, highlighted by paxillin, following fixation and permeabilization of ß1 integrin siRNA treated cells ( Additional file 6: Figure S4). These results were further confirmed by cell surface biotinylation experiments which illustrated increased cell surface biotinylation of αvß3 in ß1 integrin siRNA treated cells (Figure 4C). Thus, the increased adhesion to TGFBI associated with suppression of ß1 integrin expression is likely due to modulation in ß3 integrin expression on the cell surface. Therefore, differences in response of ovarian cancer cells to distinct ECM components may occur, dependent on their ß1/ß3 integrin expression status.

### Suppression of Syndecan-1 expression synergizes with the suppression of ß1 integrin expression to stimulate SKOV3 adhesion to rTGFBI

In addition to the integrin-family of receptors, other co-receptors are required for extracellular matrix adhesion and integrin activation [41]. One such group is the syndecan family of cell surface receptors, which have a primary role in synergizing with integrins to promote ECM binding [42]. We next determined if the most relevant syndecan members, Syndecan-1 and −4, could modulate adhesion to rTGFBI and whether they influenced the integrin cross-talk that occurs after alteration of integrin expression. SKOV3 cells stably expressing either non-target control shRNA or ß1 integrin shRNA were transfected with siRNA SMARTpool targeted against Syndecan-1. Flow cytometric analysis was performed to verify suppression of ß1 integrin in addition to suppression of Syndecan-1 protein expression (Figure 4A). Loss of both ß1 integrin and Syndecan-1 expression were synergistic in increasing adhesion of SKOV3 cells to recombinant TGFBI. By contrast, loss of Syndecan-1 expression alone had a negative effect on adhesion to recombinant periostin (Figure 4B). Furthermore, cell surface biotinylation experiments revealed increased cell surface localization of αvß3 integrin in ß1 integrin and SDC-1 single and double knockdown treated cells (Figure 4C). Suppression of Syndecan-4 expression alone in these cells had little effect and did not synergize with the loss of ß1 integrin expression to stimulate adhesion to recombinant TGFBI (Figure 4D). However, we did observe a significant suppression of adhesion to periostin after knockdown of Syndecan-4 expression (Figure 4D). Therefore, Syndecan-1 and −4 expression is dispensable for adhesion of ovarian cancer cells to rTGFBI, however, the loss of Syndecan-1 expression can synergize with the loss of ß1 integrin expression to stimulate rTGFBI adhesion.

### Unlike periostin, the carboxy-terminus of rTGFBI supports adhesion of ovarian cancer cells and is dependent on an intact RGD motif

**Figure 5:**
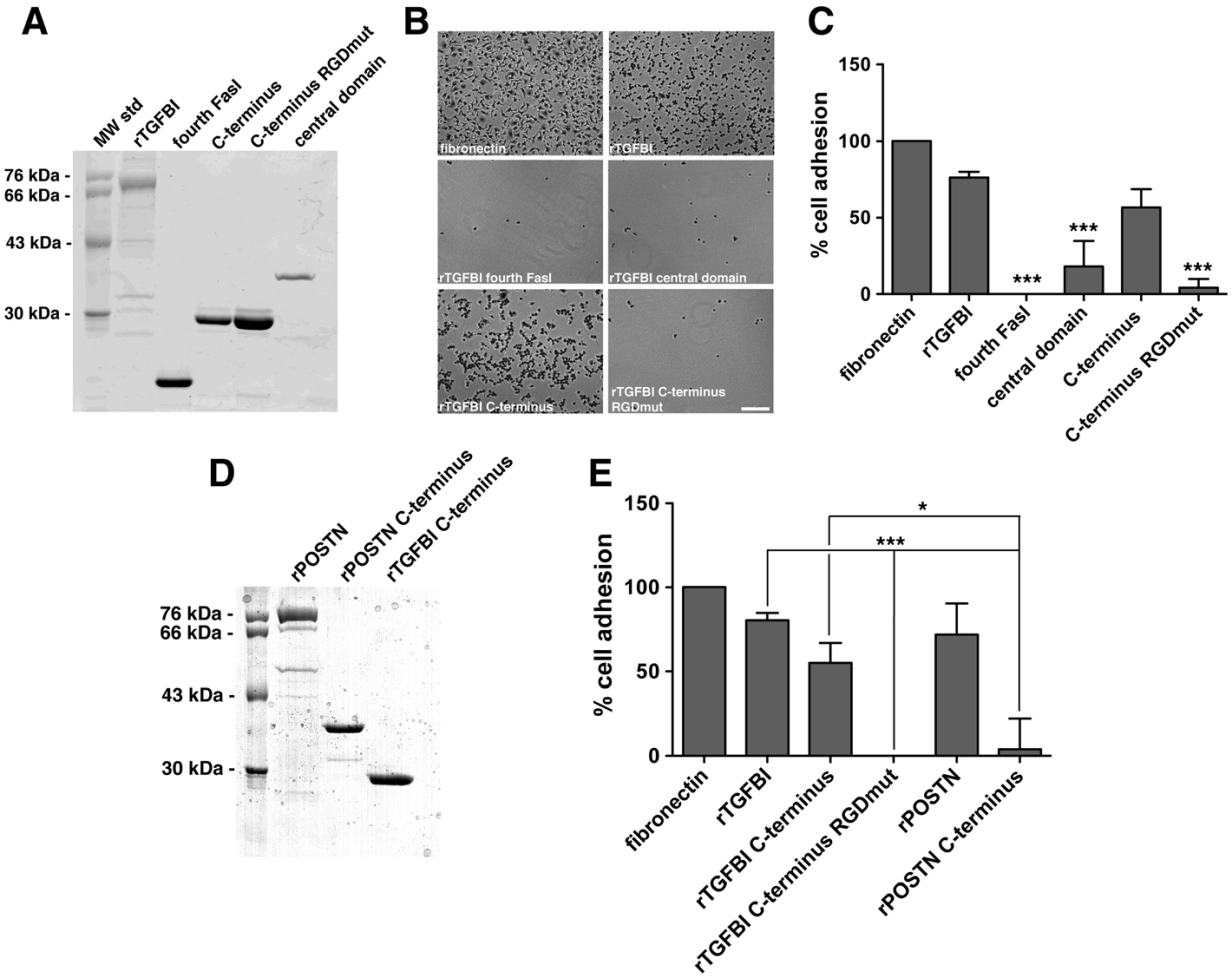
**Unlike periostin, the carboxy-terminus of rTGFBI supports adhesion of ovarian cancer cells and is dependent on an intact RGD motif.A,** Coomassie brilliant blue stained SDS-PAGE of full-length and various truncated constructs of rTGFBI purified from bacteria. **B,** SKOV3 cells replated on tissue culture wells either uncoated or coated with poly-L-lysine, fibronectin and various rTGFBI constructs for 30 minutes. Bright-field images (a-f) were processed following Giemsa staining. rTGFBI comprises aa 1–683, fourth FasI comprises aa 497–637, C-terminus comprises aa 497–683, and central domain comprises aa 24–506. Scale bar = 400 μm. **C,** Adhesion results of three independent experiments were normalized to poly-L-lysine and represented as percent of fibronectin control. Significance of ***p < 0.001 when comparing fourth FasI, central domain, and C-terminus RGDmut to full-length TGFBI. **D,** Coomassie stained SDS-PAGE of purified recombinant full-length and C-terminus of periostin along with C-terminus of TGFBI. **E,** SKOV3 cells were replated on tissue culture wells coated with indicated constructs and allowed to adhere for 30 minutes. Results are of three independent experiments, normalized to poly-L-lysine, and represented as percent of fibronectin control, *p < 0.05, ***p < 0.001.

The specificity of TGFBI for distinct integrin heterodimers may be dictated by different protein binding motifs as compared to those within periostin [6]. Recombinant truncated TGFBI constructs were produced and purified from bacteria to test which motifs were required for adhesion of SKOV3 cells (Figure 5A). The carboxy-terminus of TGFBI (aa 498–683), which contains the fourth fasciclin I domain and the RGD motif, was capable of supporting SKOV3 cell adhesion similar to full-length rTGFBI. However, the fourth fasciclin I domain alone (aa 498–637), previously shown to support HUVEC and human fibroblast cell adhesion [15,43], and the central domain (aa 24–506) were unable to support SKOV3 adhesion (Figure 5B5C). Furthermore, mutagenesis of the RGD motif to amino acid residues RAE in the carboxy-terminal truncated form of TGFBI (aa 498–683) abrogated adhesion of SKOV3 cells (Figure 5B5C).

As the carboxy-terminus of periostin includes the fourth fasciclin domain, but not a RGD motif, we asked if this region was sufficient for adhesion. Therefore, SKOV3 cells were subjected to an adhesion assay on bacterially expressed recombinant TGFBI and periostin that each comprise the fourth fasciclin I domain through to the end of the protein sequence (Figure 5D). The carboxy-terminus of periostin was unable to support cell adhesion in contrast to TGFBI (Figure 5E).

### The RGD motif of TGFBI is necessary, but not sufficient, for adhesion of ovarian cancer cells expressing ß3 integrin

**Figure 6:**
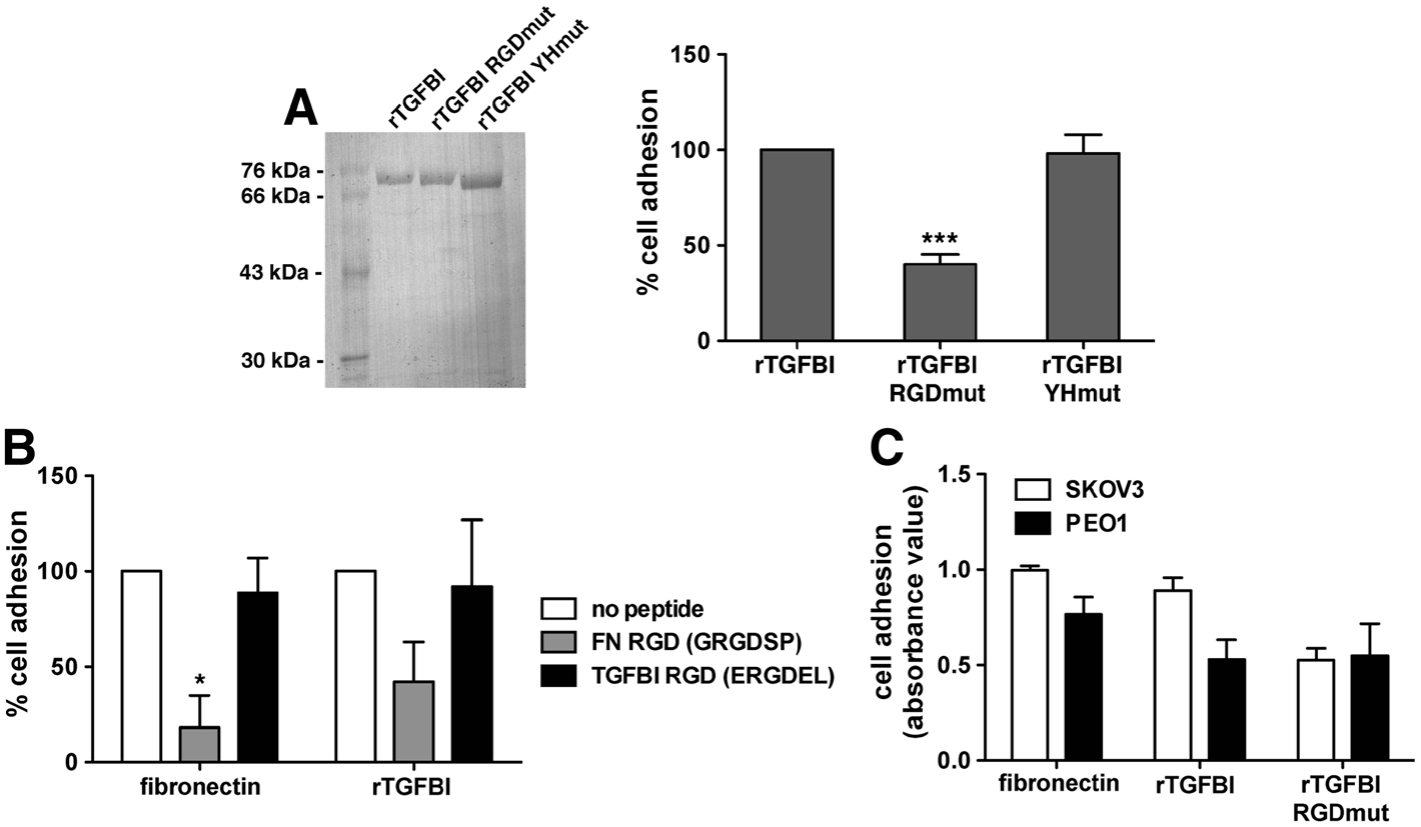
**The RGD motif of TGFBI is necessary, but not sufficient, for adhesion of ovarian cancer cells expressing ß3 integrin.A,** Coomassie stained SDS-PAGE of purified wild-type, RGDmut, or YHmut of full-length recombinant TGFBI protein from bacteria. SKOV3 cells were replated on tissue culture wells coated with indicated constructs and allowed to adhere for 30 minutes. Results of three independent experiments were normalized to poly-L-lysine and represented as percent of fibronectin control. Significance of ***p < 0.001 is compared to full-length rTGFBI. **B,** SKOV3 cells pre-incubated with either the fibronectin RGD peptide (GRGDSP) or the TGFBI RGD peptide (ERGDEL) were replated on tissue culture wells coated with fibronectin or rTGFBI. Results of two independent experiments are represented as percent of control without peptide. Significance of *p < 0.05 is compared to no peptide control. **C,** Adhesion assays were performed with SKOV3 and PEO1 cells plated on poly-D-lysine, fibronectin, rTGFBI, or rTGFBI RGDmut. Results of two independent experiments were normalized to adhesion on poly-D-lysine and are represented as relative absorbance at 540 nm.

To further understand how the fourth fasciclin I domain and the RGD motif cooperate with other TGFBI domains, we evaluated whether mutation of the RGD motif to amino acid residues RAE would affect the ability of full-length TGFBI to support SKOV3 adhesion. In these experiments we found that the RGD to RAE mutation in full-length TGFBI significantly reduced SKOV3 adhesion (Figure 6A). While mutation of the YH motif in the fourth Fasciclin I domain, previously shown to be necessary for avß3 integrin-mediated adhesion of HUVEC cells [15], did not affect cell adhesion (Figure 6A).

Short RGD peptides derived from fibronectin have been previously reported to function as inhibitors of fibronectin adhesion and migration [44,45]. Therefore, we tested whether the ERGDEL peptide derived from TGFBI was capable of competitively inhibiting adhesion of ovarian cancer cells to fibronectin and rTGFBI. Pretreatment of cells with the classical fibronectin GRGDSP peptide was capable of inhibiting adhesion to both fibronectin and rTGFBI (Figure 6B). By contrast, pretreatment with the TGFBI ERGDEL peptide did not alter adherence to fibronectin and rTGFBI (Figure 6B). Therefore, the RGD motif of TGFBI is necessary, but is not sufficient, to support adhesion of SKOV3 cells and binding either requires a greater number of flanking amino acids or a complex with the fourth Fasciclin I domain.

This may be further modulated by the integrin expression profile that dictates the mechanism by which TGFBI interacts with the cell surface, as PEO1 cells, which lack ß3 integrin, do not require the RGD motif of TGFBI for adhesion (Figure 6C). This is in contrast to the SKOV3 cell line, which requires the RGD motif of TGFBI for maximal adhesion (Figure 6A, 6C). Therefore, although ovarian cancer cells have the ability to adhere to both periostin and TGFBI, they likely utilize distinct mechanisms.

### Suppression of different integrin and ECM components has distinct effects on paclitaxel-induced death in ovarian cancer cells

**Figure 7:**
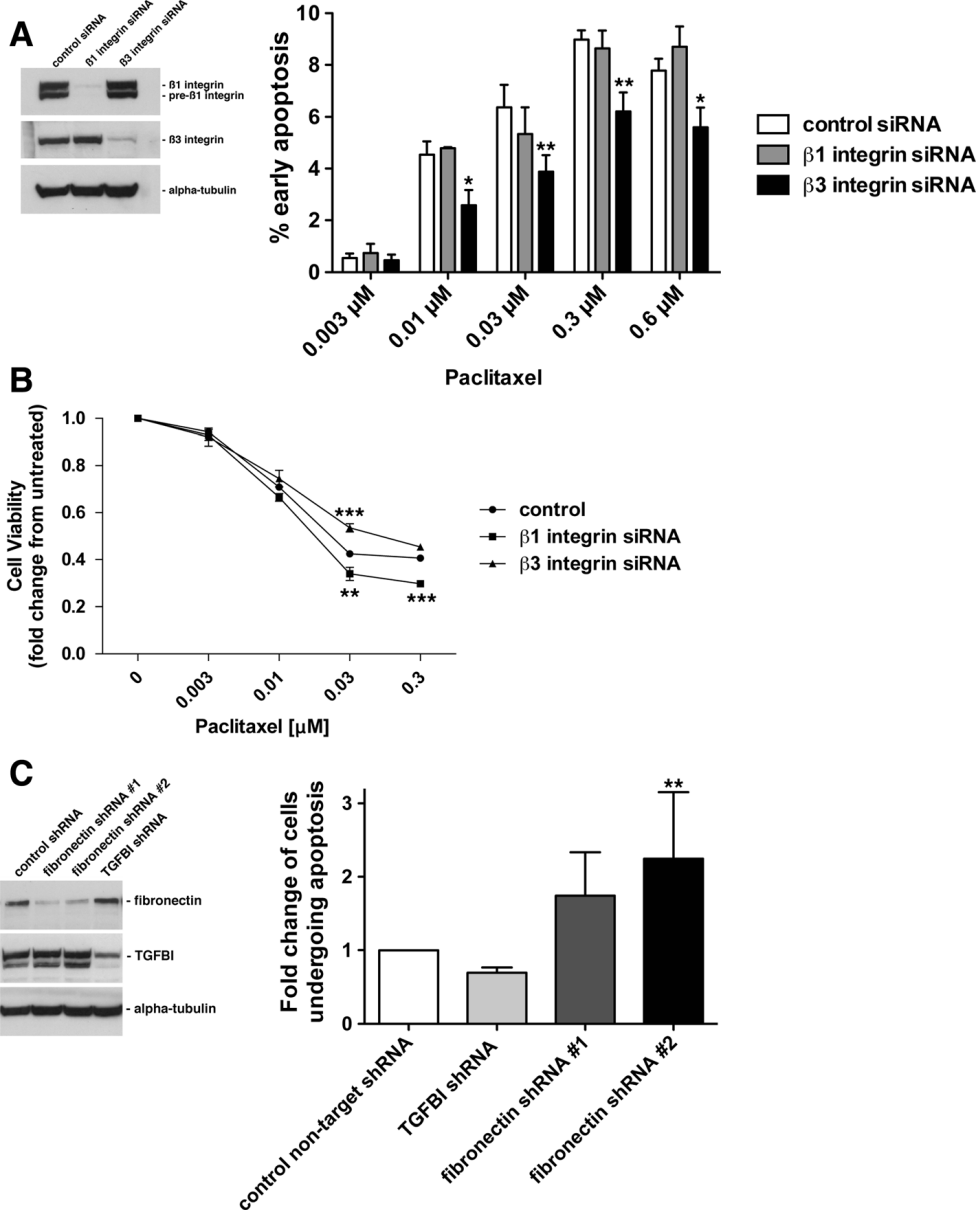
**Suppression of different integrin and ECM components has distinct effects on paclitaxel-induced death in ovarian cancer cells.A,** Western blot analysis was performed on SKOV3 cells transfected with either non-target control siRNA, ß1 integrin siRNA, or ß3 integrin siRNA. Cells were subjected to varying concentrations of paclitaxel for 24 hours followed by flow cytometric analysis of Annexin-V and propidium iodide staining, *p < 0.05, **p < 0.01. **B,** Cell titre glo cell viability assay performed on non-target control, ß1 integrin, or ß3 integrin siRNA expressing cells, treated with increasing concentrations of Paclitaxel for 48 hours. **p < 0.01, ***p < 0.001. **C,** SKOV3 cells were infected with Lentivirus expressing either non-target control shRNA, TGFBI shRNA, or two separate fibronectin shRNA targets. Western blot analysis was performed on RIPA soluble lysates using antibodies to the specified proteins. Cells were treated with 0.3 μM paclitaxel for 24 hours followed by Annexin-V and propidium iodide staining, and subsequently analyzed by flow cytometry. Experiments were performed in triplicate and are represented as fold increase in cells in early apoptosis, **p < 0.01.

Integrin-mediated signaling has been suggested to influence the cytotoxic effects of paclitaxel on cancer cells [1,30,46]. We have previously shown that loss of TGFBI expression subsequently leads to cells becoming resistant to paclitaxel-induced cell death, dependent on ß3 integrin function [1]. Studies in breast cancer cells indicated that fibronectin-mediated and ß1 integrin-dependent signaling was required for a paclitaxel resistant phenotype. Therefore, we directly tested whether there was specificity among different integrin heterodimers that dictated the response of cells to paclitaxel. We used siRNA to suppress ß1 and ß3 integrin expression in SKOV3 cells, and evaluated response to paclitaxel-induced death. Importantly, compared to control, loss of ß3 integrin expression induced a partial paclitaxel-resistant phenotype, as shown by a decrease in apoptosis and an increase in cell viability, while the loss of ß1 integrin expression had no effect on apoptosis and a partial decrease in cell viability, suggesting a minor paclitaxel-sensitive phenotype, consistent with previous reports [30] (Figure 7A7B). Therefore, our data suggest that discrete signaling pathways may exist downstream of ß1 and ß3 integrin activation that influence the response of cells to paclitaxel induced death, which may provide a unique role for ß3 integrin-specific ECM proteins, such as TGFBI, in this process. This is further supported by the loss of TGFBI expression leading to a paclitaxel resistant phenotype, while suppression of fibronectin expression, preferentially signaling through ß1-integrin, inducing a paclitaxel sensitive phenotype (Figure 7C). Therefore, deregulation of distinct integrin-mediated signaling pathways may have contrasting effects on paclitaxel response.

## Discussion

TGFBI is a multifunctional protein implicated in a variety of physiological processes including cell growth, wound healing, inflammation, and developmental morphogenesis [6]. However, its dysregulation can lead to the pathogenesis of a variety of diseases, including cancer [6]. More specifically, recent evidence suggests that TGFBI is dysregulated in ovarian cancer and its expression level may influence cancer response to the chemotherapeutic agent paclitaxel [1]. In addition, extracellular TGFBI increases the motility and invasiveness of ovarian cancer cells and stimulates a peritoneal cell interaction [47]. Therefore, we sought to understand the molecular mechanisms that influence TGFBI function and its interrelationship with other ECM components known to be present in the tumor microenvironment in order to better determine potential therapeutic targets and indicators of treatment response.

In ovarian cancer cells, which express both the ß1 and ß3 integrin subunits, TGFBI preferentially interacts with cells through an αvß3 integrin-mediated mechanism. This is in contrast to the predominant ß1 integrin-mediated mechanism elicited by fibronectin and periostin (Figure 2C). Although this contradicts recent evidence that suggests periostin primarily interacts with ovarian cancer cells via an αvß3 integrin-dependent mechanism [24], it also suggests a delicate balance may exist between different integrin receptors on the cell surface that dictate specificity to the ECM. This is further supported by our data showing that loss of ß1 integrin in SKOV3 cells increases adhesion to rTGFBI, but not to fibronectin or periostin, in an αvß3 integrin dependent manner (Figure 3; Additional file 5: Figure S3).

Additionally, integrin cross-talk may play a major role in the diversity seen within different cell systems and within different tumor types that have varying integrin subunit expression profiles. For example, divergent signaling through ß1 and ß3 integrins has major impacts on downstream Rho GTPase signaling, which may subsequently result in contrasting effects on cell adhesion and migration [48]. In addition, distinct ß1 and ß3 integrin expression along with oncogene expression, such as oncogenic Src, may differentially influence chemosensitivity [49]. Our data supports this notion as suppression of ß1 integrin expression stimulates a TGFBI-ß3 integrin-mediated adhesion response (Figure 3). Although our data suggests an increased cell surface expression of the αvß3 integrin heterodimer following suppression of ß1 integrin expression (Figure 3E4C), there likely also exists cross-talk between downstream signaling complexes associated with the activation of different integrin receptors. Furthermore, our data indicate that in ovarian cancer cells the loss of ß3 integrin expression partially induces a paclitaxel-resistant phenotype, while loss of ß1 integrin expression leads to a potential paclitaxel-sensitive phenotype. With regards to integrin receptor cross-talk, it has been previously reported that forced expression of α5ß1 integrin negatively regulates αvß3 integrin function in Chinese hamster ovary cells [31]. In addition, it has been shown, with regards to the α3 noncollagenous domain of collagen IV, that the α3ß1 heterodimer can modulate αvß3-mediated cell adhesion [32]. Lastly, ß1 integrin activation negatively effects αvß3 activation via activation of PKA and inhibition of PP1 activity [33]. Since ß3 integrin expression has been suggested to be a potential prognostic biomarker in ovarian cancer [50], it will be important to delineate the specific ß3 integrin-dependent signals and determine their impact on ovarian carcinogenesis and chemotherapy response.

Attachment of ovarian cancer to the mesothelium and its associated ECM, which lines the peritoneal cavity, may not be exclusively integrin-mediated [38,51]. Therefore, other integrin-independent cell-ECM receptors may be involved in mediating adhesion in the tumor microenvironment. The primary co-receptor family involved in cell-ECM adhesion, which synergizes with integrin engagement to mediate a complete cellular response, is the Syndecan family of receptors [42]. For αvß3 adhesion, Syndecan-1 is the predominant co-receptor that mediates this process [52]. It is important to note that although Syndecan-1 expression is absent in the normal ovary, it is upregulated in ovarian cancer as well as in tumor stroma [53]. Our data suggests that the loss of Syndecan-1 cooperates with the loss of ß1 integrin to stimulate adhesion to TGFBI and is therefore dispensable for TGFBI adhesion. Thus, for ovarian cancer cells, it appears neither Syndecan-1 nor Syndecan-4 is necessary for adhesion to TGFBI, nor does the loss of Syndecan-4 synergize with αvß3 to stimulate adhesion to TGFBI. In contrast, for periostin, loss of both Syndecan-1 and Syndecan-4 negatively affects ovarian cancer cell adhesion, which supports the notion that periostin utilizes a distinct mode of cellular interaction.

Previous literature has attempted to dissect the specific domains and motifs within TGFBI that are critical for its interactions with the cell surface. Since these results seem to be cell-type and system specific, we attempted to extend a similar analysis with respect to ovarian cancer cells, including the comparison to its paralogue, periostin, which has been suggested to have a proactive role in ovarian cancer migration [24]. Importantly, unlike TGFBI, the periostin carboxy-terminus, which lacks an RGD motif, is unable to support adhesion. This suggests that the specificity of TGFBI and periostin for their respective cell surface receptors is partially dictated by differences in this region.

The function of the TGFBI-derived RGD appears to vary depending on the cell-based system and the primary integrin heterodimeric receptor. Initially, it was suggested that the carboxy-terminus underwent proteolytic processing resulting in loss of the RGD motif [54,55]. In addition, this carboxy-terminal released peptide induced apoptosis in CHO cells is dependent on an intact RGD motif [55]. However, *in vitro* biochemical analysis suggested the potential carboxy-terminal cleavage site on human TGFBI lies downstream of the RGD motif suggesting it is not cleaved from the full-length protein [56]. Therefore, a mature TGFBI protein that contains the RGD motif is likely functional in a biological context. This is supported by recent data that suggests the RGD motif of TGFBI is necessary for promoting extravasation of metastatic colon cancer cells [16]. Our results suggest that the RGD motif of full-length TGFBI is necessary, but not sufficient, for ovarian cancer cell adhesion, thus indicating it may cooperate with flanking residues or other motifs, potentially present within the fourth Fasciclin I domain to mediate this process. Importantly, we found that the TGFBI derived RGD peptide (ERGDEL) was unable to competitively inhibit SKOV3 adhesion to rTGFBI, suggesting its use as a therapeutic agent to inhibit TGFBI function may depend on the cellular context.

## Conclusions

Ovarian cancer is a complex disease where the tumor microenvironment plays an active role in the dissemination of the disease and influences the response to chemotherapy. Since it has previously been shown that fibronectin-mediated ß1 integrin signaling represses paclitaxel-induced cell death [30], specific ECM-receptor pathways may be important in differentially modulating chemotherapeutic response. This is confirmed by our data which showed suppression of fibronectin expression sensitizes cells to paclitaxel-induced death, while suppression of TGFBI leads to a resistant phenotype (Figure 7) [1]. This is further supported by recent data in non-small cell lung cancer showing that TGFBI-mediated induction of apoptosis in response to chemotherapy requires the αvß3 integrin receptor [57]. In addition, distinct ECM-integrin receptor engagement may trigger intracellular cues that stabilize the microtubule cytoskeleton, which has been suggested to be a mechanism to enhance the cytotoxicity of paclitaxel [58]. Therefore, it will be crucial in a clinical context to define the relationship between discrete integrin heterodimers and their respective extracellular binding partners in order to understand the intracellular signaling pathways that occur. Importantly, further characterization of the differential signaling downstream of TGFBI-ß3 integrin engagement in comparison to other ECM-receptor mediated pathways will be needed to identify distinct mechanisms of chemotherapeutic response.

## Competing interests

The authors declare they have no competing interests.

## Authors’ contributions

DAT designed and performed experiments, analysed the data, and wrote the manuscript. JT performed the cell titre glo cell viability assay. JDB conceived of the study and wrote the manuscript. All authors read and approved the final manuscript.

## Supplementary Material

Additional file 1 **Movie S1.** Bright-field time lapse video microscopy of an SKOV3 cell plated on rTGFBI in serum-free media. Images were acquired every 2 minutes for a period of 6 hours. Click here for file

Additional file 2 **Movie S2.** Bright-field time lapse video microscopy of an SKOV3 cell plated on fibronectin in serum-free media. Images were acquired every 2 minutes for a period of 6 hours. Click here for file

Additional file 3 **Figure S1.** Flow cytometric analysis of SKOV3, PEO1, and TR125 cell surface integrin expression following immunostaining with either IgG control or integrin-specific antibodies (P5D2 - β1, LM609 - αvβ3, P1F6 - αvβ5). Click here for file

Additional file 4 **Figure S2.** PEO1 cell adhesion to fibronectin, rTGFBI, rPOSTN and vitronectin coated tissue culture plastic in the presence of vehicle or the indicated integrin receptor blocking antibodies. Results are represented as the mean of two independent experiments normalized to uncoated and poly-L-lysine coated wells and represented as percent of vehicle treated control. Error bars represent the standard deviation. Click here for file

Additional file 5 **Figure S3.** SKOV3 cells expressing either control shRNA or β1 shRNA were left untreated or pretreated with the αvβ3 blocking antibody, LM609, prior to replating on rTGFBI. Relative adhesion was quantitated by measuring the absorbance at 540 nm and values were normalized to an uncoated well. Results represent 3 independent experiments and error bars represent standard deviation. *p < 0.05, **p < 0.001. Click here for file

Additional file 6 **Figure S4.** Immunofluorescence microscopy of SKOV3 cells stably expressing control or β1 integrin shRNA. Fixed and permeabilized cells were immunostained for paxillin, to highlight focal adhesions, and the actin cytoskeleton was visualized with Alexa Flour 568-phallloidin. Scale bar 40 μm. Click here for file
