# Supplementary figures and images for: ß3 integrin modulates transforming growth factor beta induced (TGFBI) function and paclitaxel response in ovarian cancer cells
Source: Mol Cancer. 2012 May 28;11:36. doi: 10.1186/1476-4598-11-36 (PMC3442987; doi:10.1186/1476-4598-11-36)

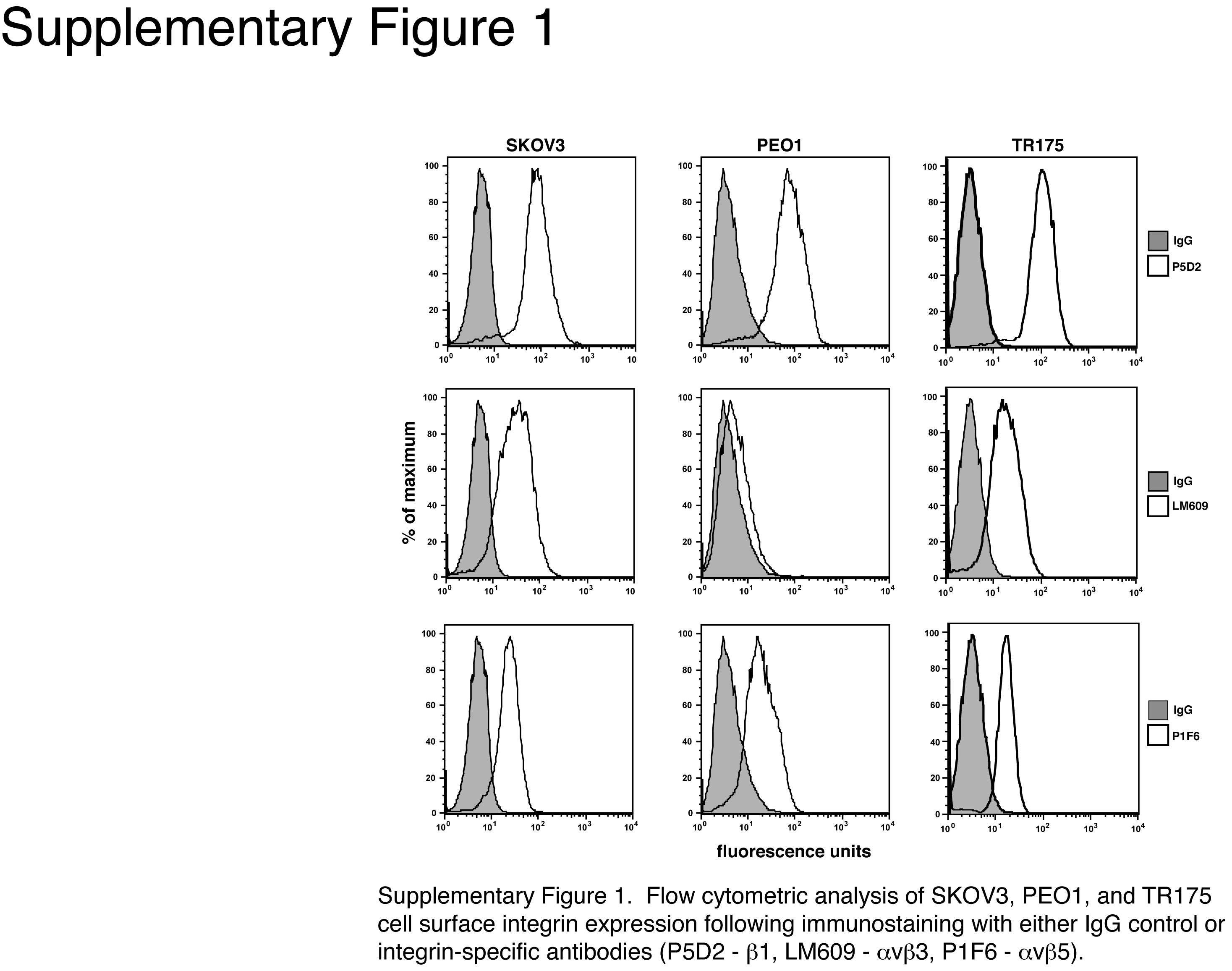

Supplement: Additional file 1 — Movie S1. Bright-field time lapse video microscopy of an SKOV3 cell plated on rTGFBI in serum-free media. Images were acquired every 2 minutes for a period of 6 hours. [file 1476-4598-11-36-S1.tiff]

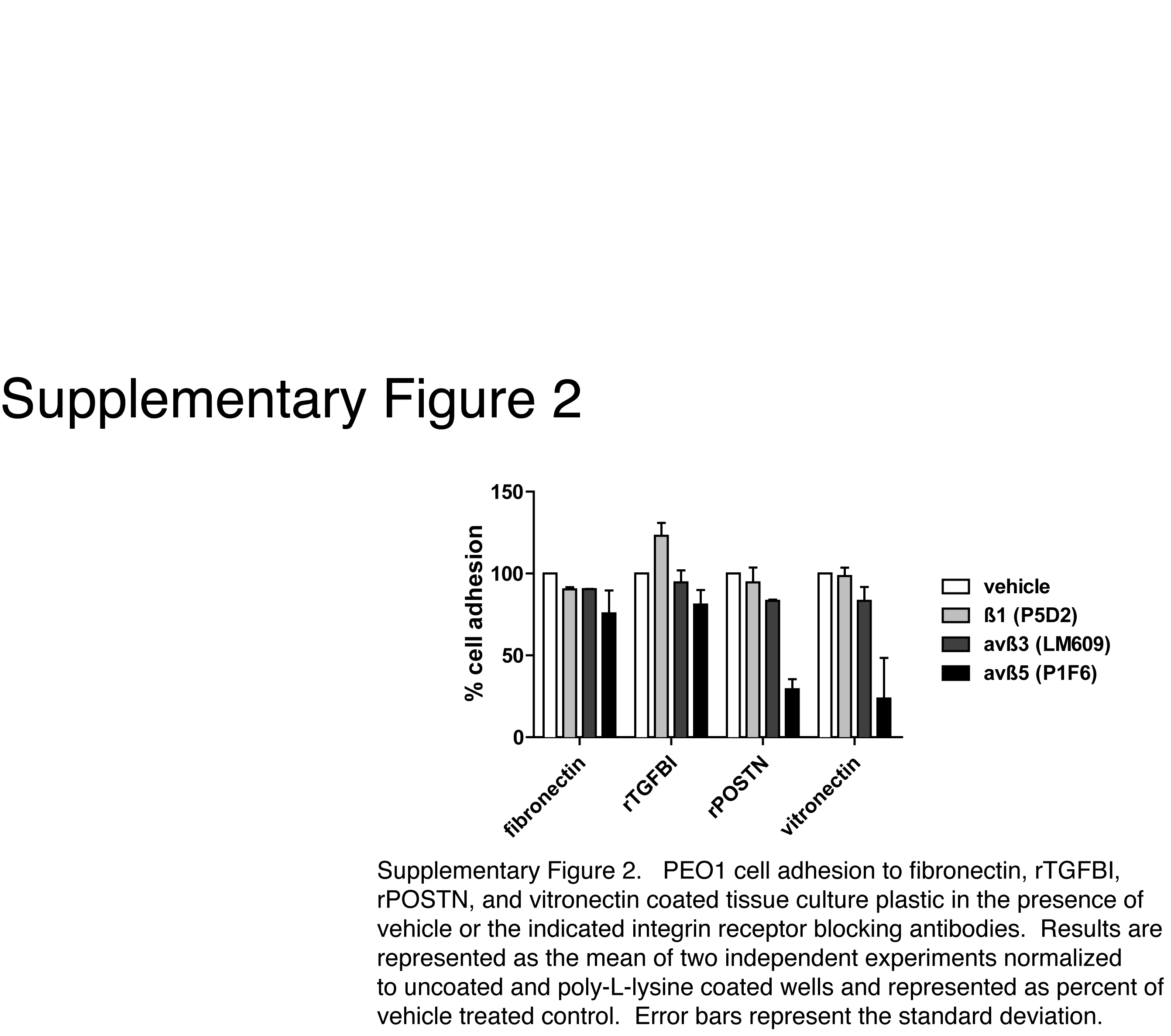

Supplement: Additional file 2 — Movie S2. Bright-field time lapse video microscopy of an SKOV3 cell plated on fibronectin in serum-free media. Images were acquired every 2 minutes for a period of 6 hours. [file 1476-4598-11-36-S2.tiff]
